# Non-invasive quantification of stem cell-derived islet graft size and composition

**DOI:** 10.1007/s00125-024-06194-5

**Published:** 2024-06-14

**Authors:** Väinö Lithovius, Salla Lahdenpohja, Hazem Ibrahim, Jonna Saarimäki-Vire, Lotta Uusitalo, Hossam Montaser, Kirsi Mikkola, Cheng-Bin Yim, Thomas Keller, Johan Rajander, Diego Balboa, Tom Barsby, Olof Solin, Pirjo Nuutila, Tove J. Grönroos, Timo Otonkoski

**Affiliations:** 1 Stem Cells and Metabolism Research Program, Faculty of Medicine, University of Helsinki, Helsinki, Finland; 2 Turku PET Centre, University of Turku, Turku, Finland; 3 Medicity Research Laboratories, University of Turku, Turku, Finland; 4 Accelerator Laboratory, Turku PET Centre, Åbo Akademi University, Turku, Finland; 5 Department of Chemistry, University of Turku, Turku, Finland; 6 Department of Endocrinology, Turku University Hospital, Turku, Finland; 7 The Wellbeing Services County of Southwest Finland, Turku, Finland; 8 Children’s Hospital, Helsinki University Hospital, Helsinki, Finland

**Keywords:** Beta cell mass, Cell replacement therapy, Congenital hyperinsulinism, PET, Positron emission tomography, Stem cell-derived islets, Transplantation, Type 1 diabetes

## Abstract

**Aims/hypothesis:**

Stem cell-derived islets (SC-islets) are being used as cell replacement therapy for insulin-dependent diabetes. Non-invasive long-term monitoring methods for SC-islet grafts, which are needed to detect misguided differentiation in vivo and to optimise their therapeutic effectiveness, are lacking. Positron emission tomography (PET) has been used to monitor transplanted primary islets. We therefore aimed to apply PET as a non-invasive monitoring method for SC-islet grafts.

**Methods:**

We implanted different doses of human SC-islets, SC-islets derived using an older protocol or a state-of-the-art protocol and SC-islets genetically rendered hyper- or hypoactive into mouse calf muscle to yield different kinds of grafts. We followed the grafts with PET using two tracers, glucagon-like peptide 1 receptor-binding [^18^F]F-dibenzocyclooctyne-exendin-4 ([^18^F]exendin) and the dopamine precursor 6-[^18^F]fluoro-l-3,4-dihydroxyphenylalanine ([^18^F]FDOPA), for 5 months, followed by histological assessment of graft size and composition. Additionally, we implanted a kidney subcapsular cohort with different SC-islet doses to assess the connection between C-peptide and stem cell-derived beta cell (SC-beta cell) mass.

**Results:**

Small but pure and large but impure grafts were derived from SC-islets. PET imaging allowed detection of SC-islet grafts even <1 mm^3^ in size, [^18^F]exendin having a better detection rate than [^18^F]FDOPA (69% vs 44%, <1 mm^3^; 96% vs 85%, >1 mm^3^). Graft volume quantified with [^18^F]exendin (*r*^2^=0.91) and [^18^F]FDOPA (*r*^2^=0.86) strongly correlated with actual graft volume. [^18^F]exendin PET delineated large cystic structures and its uptake correlated with graft SC-beta cell proportion (*r*^2^=0.68). The performance of neither tracer was affected by SC-islet graft hyper- or hypoactivity. C-peptide measurements under fasted or glucose-stimulated conditions did not correlate with SC-islet graft volume or SC-beta cell mass, with C-peptide under hypoglycaemia having a weak correlation with SC-beta cell mass (*r*^2^=0.52).

**Conclusions/interpretation:**

[^18^F]exendin and [^18^F]FDOPA PET enable non-invasive assessment of SC-islet graft size and aspects of graft composition. These methods could be leveraged for optimising SC-islet cell replacement therapy in diabetes.

**Graphical Abstract:**

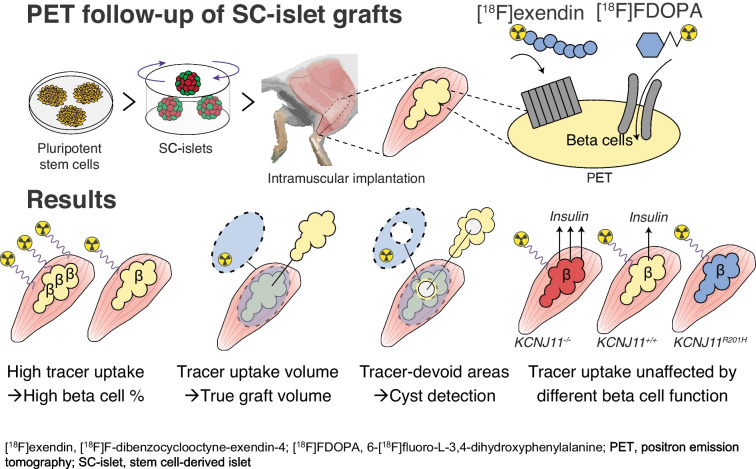

**Supplementary Information:**

The online version contains supplementary material available at 10.1007/s00125-024-06194-5.


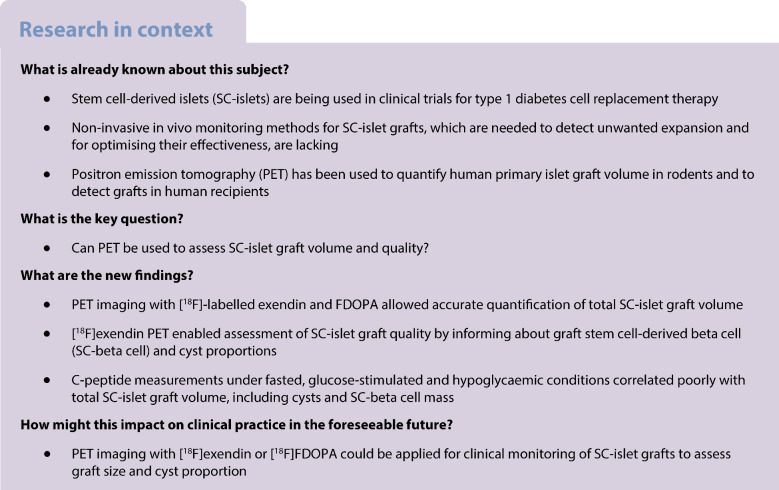


## Introduction

Islet or pancreas transplantation remains the only route to independence from exogenous insulin for individuals with type 1 diabetes. Islet transplantation is limited by the scarcity of cadaveric islets, as usually at least two donors are required per individual [1]. Functional islets differentiated in vitro from pluripotent stem cells (stem cell-derived islets [SC-islets]) [2–5] on the other hand are abundant, are functionally consistent and can feasibly be genome-edited or encapsulated to circumvent the need for systemic immunosuppression [6, 7]. Given these advantages, SC-islet-based cell replacement therapy has the potential to revolutionise the treatment of type 1 diabetes [8]. SC-islet therapy has enabled insulin independence in a few individuals with type 1 diabetes after intraportal SC-islet implantation (ClinicalTrials.gov registration no. NCT04786262) [9] and additional trials are ongoing (NCT05791201, NCT05565248) with different strategies.

Nonetheless, clinical use of stem cell-derived tissue has inherent risks. Lingering progenitor cells can differentiate in vivo to undesired pancreatic ductal cysts [10–16], potentially hampering therapeutic effectiveness. Furthermore, in vitro culture conditions can enrich chromosomal abnormalities and oncogenic mutations in pluripotent stem cells [17, 18]. If needed, the SC-islet graft can be eliminated by cessation of immunosuppressants, unless the graft is autologous or immune-evasive. Engineered immune evasion of SC-islets is under preclinical [4, 19–23] and clinical (NCT05565248) development. Immune evasion would facilitate invasion and metastasis of any neoplasm originating from the edited cells [6, 23, 24]. In an extreme demonstration of these risks, an implant of autologous stem cell-derived cells claimed to have been differentiated into SC-islets gave rise to a metastatic teratoma in 2 months [25].

Given the enormous promise and the potentially substantial risks of SC-islet cell replacement therapy, a robust non-invasive method for in vivo monitoring is needed. Optimally, it would (1) quantify graft size; (2) quantify graft composition (e.g. beta cell mass); and (3) not be affected by beta cell functionality. Improved monitoring of SC-islet grafts could be used to assess the in vivo impact of, for example, improved differentiation protocols, different implantation sites and survival improvement strategies [22, 26–31]. Detecting graft size increase or compositional change could inform immunosuppression cessation or suicide switch activation [32, 33].

Current non-invasive longitudinal monitoring methods rely mostly on C-peptide measurements. C-peptide measurements inform about beta cell function, but give little information about overall graft composition, and it is unclear how well C-peptide measurements quantify engrafted SC-islet volume.

Positron emission tomography (PET), an imaging technique employing target-specific radiotracers and the most sensitive imaging modality in humans, has long been developed for quantifying beta cell mass in the native pancreas and in primary islet grafts [34, 35]. In the primary islet transplantation context, PET and other nuclear imaging methods have been shown to enable detection of human islet grafts in human muscle [36] and liver [37, 38], as well as quantifying human islet graft size of grafts transplanted in rodent muscle [39, 40]. Many of these studies used tracers targeting the glucagon-like peptide 1 receptor (GLP1R), expressed in human pancreas primarily in beta cells [41, 42]. In light of this, we recently optimised ^18^F-labelling of the GLP1R ligand exendin-4 ([^18^F]F-dibenzocyclooctyne-exendin-4, referred to as ‘[^18^F]exendin’ throughout this article) [43]. Imaging the pancreas with ^18^F-labelled 6-fluoro-l-3,4-dihydroxyphenylalanine (DOPA) ([^18^F]FDOPA), a dopamine precursor analogue, is established in the diagnosis of the focal form of congenital hyperinsulinism (CHI) [44]. Focal CHI presents with an adenomatous lesion with hypersecretory beta cells, which has a high uptake of [^18^F]FDOPA, different from other pancreatic areas and enabling precise lesionectomy [45]. Furthermore, uptake of [^18^F]FDOPA can detect human primary islet grafts in mice [46], and the uptake of the metabolically similar tracer ^11^C-5-hydroxy-tryptophan ([^11^C]HTP) can detect them also in human liver [38]. Graft monitoring of SC-islet-derived grafts specifically was recently explored with ^18^F-labelled anti-DiGeorge syndrome critical region gene 2 (DGCR2) antibody PET, which allowed transient visualisation of grafts in mice, but the method’s suitability for quantifying graft size and composition was not demonstrated [47].

Given the lack of methods established for SC-islet graft monitoring, we aimed to apply PET as a non-invasive monitoring method for SC-islet grafts suitable for clinical use. Specifically, we aimed to investigate the relationships of SC-islet graft volume, composition and functional state with the uptake of the PET tracers [^18^F]exendin and [^18^F]FDOPA and compare their performance with C-peptide measurements.

## Methods

### Stem cell lines and SC-islet differentiation

Mutation-corrected induced pluripotent cell line HEL113.5 [48]-derived SC-islets were used for cohort-1. They were differentiated according to the Balboa et al 2018 protocol, modified to use Aggrewell 400 plates (Stem Cell Technologies, Canada) for aggregation [49]. SC-islets differentiated from the H1 embryonic stem cell line (WiCell, USA) and its gene-edited counterparts according to the Barsby et al 2022 protocol were used for cohort-2 [50]. The stem cell lines tested negative for mycoplasma spp.

### Genome editing of the *KCNJ11* locus

The H1 cell line was separately genome-edited to generate *KCNJ11* knockout and *KCNJ11* R201H knockin clones using the ribonucleoprotein CRISPR-Cas9 system (electronic supplementary material [ESM] Methods).

### SC-islet implant volume determination

Implant preparation volume was determined by hand-picking SC-islets for each graft (picked after the first week of stage 7 when using the Balboa et al 2018 protocol [49] and after the fifth or sixth week of stage 7 when using the Barsby et al 2022 protocol [50]) and taking an image of them, which was analysed using a custom CellProfiler pipeline (version 3.0) [51]. This reported the number and median diameter of the SC-islets for each graft, from which the total implant volume could be calculated. SC-islet doses between 1 and 6.3 mm^3^ of SC-islets were used in the study. This corresponds to 566–3566 islet equivalents (IEQs, spherical islets with 150 µm diameter [52], each 0.001767 mm^3^) or around 150–950 SC-islets (median 230 µm diameter). In cohort-1, four doses were used (volumes in mm^3^)—around 1.3, 2.5, 3.8 or 5.0—with the left calf implanted with a smaller graft than the right. In cohort-2, doses of either 3 or 5 mm^3^ of SC-islets were implanted in both legs. The left leg was implanted with *KCNJ11*^−/−^ or *KCNJ11*^+/R201H^ SC-islets and the right leg with *KCNJ11*^+/+^ SC-islets. Sizes for the kidney cohort were around 1, 3 or 6.3 mm^3^. This predetermined implant volume was then aspirated with a precision syringe (Hamilton, USA, no. 81301) into silicone tubing, compacted into a pellet by 2 min of centrifugation at 100*g* and kept on ice until implantation.

### SC-islet implantation

Two- to five-month-old 20–30 g immunocompromised female NOD-*scid*-gamma mice (The Jackson Laboratory, USA; no. 005557, www.jax.org/strain/005557) were randomly allocated for different types of implants, anesthetised with isoflurane (5% induction, 2% maintenance) and shaved to expose the calves. Tubing containing the SC-islet pellet was connected to a precision syringe at one end and a 0.6 mm needle (Sarstedt, Germany, no. 85.923) at the other end. The needle was inserted into the musculus gastrocnemius through the distal, posterolateral calf and pushed approximately 5 mm inside. The SC-islets were implanted while retracting the needle by approximately 3 mm. After implantation, if any SC-islets remained inside the opaque needle, their volume was quantified and subtracted from the implanted volume. Kidney implantations were conducted on female mice as previously reported [2].

### Animal husbandry and C-peptide measurements

After implantation the mice were fed irradiated standard chow ad libitum and kept on a 12 h dark/12 h light cycle. Insulin and glucose tolerance tests were conducted as previously described [2, 48], with further details in the ESM Methods.

### Radiotracer synthesis

Radiotracers were synthetised in the Turku PET Centre radiochemistry laboratory, University of Turku. [^18^F]FDOPA was synthesised via electrophilic ^18^F-fluorination as previously described [53]. [^18^F]F-dibenzocyclooctyne-exendin-4 (Nle^14^,Cys^40^(Mal-dPEG4-DBCO-N_3_-PEG3-ethyl[^18^F]fluoro-exendin-4)) was synthesised in two steps using a prosthetic group strategy [43]. In the first step the prosthetic group, azido-PEG3-ethyl[^18^F]fluoride, was synthesised from tosylate precursor and K_222_/[^18^F]/K^+^-complex via nucleophilic fluorination. In the final step, [^18^F]F-dibenzocyclooctyne-exendin-4 ([^18^F]exendin) was synthetised from cyclooctyne-derivatised exendin-4 and azido-PEG3-ethyl[^18^F]fluoride via strain-promoted azide–alkyne cycloaddition. The [^18^F]exendin was purified via semipreparative HPLC and formulated in 10% ethanol in 0.1 mmol/l PBS containing 0.02% ascorbic acid. Molar activity of [^18^F]exendin was 40–100 and [^18^F]FDOPA >3 GBq/µmol at the end of synthesis.

### PET/computed tomography imaging

Mice were fasted for 4 h prior to imaging to standardise blood glucose levels. Imaging was conducted in the early afternoon. One or two mice were scanned at a time, with small-animal PET/computed tomography (CT) (X- and β-cubes, Molecubes, Belgium) carried out after i.v. administration of approximately 2–3 MBq of either [^18^F]FDOPA or [^18^F]exendin under isoflurane anaesthesia. A 10 min CT scan for attenuation correction and anatomical reference preceded PET. Dynamic PET data were collected for 30 min, divided into 25 time frames (12 × 10 s, 6 × 30 s, 5 × 60 s, 2 × 600 s) in list mode and reconstructed with an OSEM3D algorithm included in the Molecubes scanner software. The radioactivity uptake was corrected for radionuclide decay and expressed as percentage of injected dose per gram of tissue (%ID/g). The scanner has a 13 cm axial field of view and a 7.2 cm transaxial field of view, generating 192 transaxial slices with voxel dimensions of 0.4 × 0.4 × 0.4 mm. PMOD software (version 4.0, PMOD Technologies, Switzerland) was used to convert reconstructed Digital Imaging and Communications in Medicine (DICOM) images produced by the β-cube into an Inveon Research Workplace (version 4.2, Siemens Medical Solutions, Germany)-compatible DICOM format for data analyses.

### PET data analysis

The image time frames were summed and scaled from 0 to 5 %ID/g for [^18^F]exendin and 0 to 7 %ID/g for [^18^F]FDOPA. Volumes of interest (VOIs) were drawn according to hotspots detected in the calf (VOI1). VOI1 inclusion was determined by contrast between background uptake and high tracer-uptake areas, as shown in the results. Areas inside the VOI1 displaying low uptake were included for all grafts. Three grafts were quantified again by excluding these low-uptake areas. Larger VOIs encompassing the entire calf muscle (VOI2) were drawn to determine background uptake. As the emission activity (*A*) inside VOI2 is composed of activity inside VOI1 and the background activity (*A*_VOI2_ = *A*_VOI1_ + *A*_Bg_) and activity is defined as the product of the uptake or tracer concentration (*C*) and the volume (*V*) (*A *= *C *× *V*), the following formula can be derived:

$${C}_{\text{Bg}} = \left({C}_{\text{VOI}2} \times {V}_{\text{VOI}2} - {C}_{\text{VOI}1} \times {V}_{\text{VOI}1}\right)/\left({V}_{\text{VOI}2} - {V}_{\text{VOI}1}\right)$$

As activity inside VOI1 is the sum of activity inside the graft and background activity (*A*_VOI1_ = *A*_Graft_ + *A*_Bg_), and the graft volume (*V*_Graft_) was determined histologically, the uptake concentration inside the graft volume can be derived. These graft uptake values correct for dilution in uptake due to the enlarged VOI1 volume:

$${C}_{\text{Graft}} = \left[{C}_{\text{VOI}1} \times {V}_{\text{VOI}1} - {C}_{\text{Bg}} \times ({V}_{\text{VOI}1} - {V}_{\text{Graft}})\right]/{V}_{\text{Graft}}$$

### Autoradiography

After the 3 month [^18^F]exendin or [^18^F]FDOPA PET scan, mice were euthanised, and their calf muscles were excised, frozen in chilled isopentane and cut into 20 μm sections (Microm HM 500 OM, Thermo Fisher, USA). The sections were exposed to an imaging plate (BAS Imaging Plate TR2025, Fuji, Japan) for 4 h and scanned using the Fuji BAS5000 analyser. Images showing the tracer uptake were later compared with insulin immunohistochemistry of adjacent sections.

### Histological examination of graft volume and cyst proportion

The total and cyst-free graft volumes were calculated using depth recorded when sectioning through paraffin-embedded graft-bearing muscle, and the total and cyst-free areas from quantified stitched images, with further details in the ESM Methods.

### Histological examination of graft composition

Immunohistochemistry was conducted and analysed essentially as in [48], with full details in the ESM Methods.

### Statistical methods

Prism version 9.5 (GraphPad software, USA) was used for statistical analyses. The *p* values for linear regression are given for the slope being non-zero. The multiple linear regression model and correlation heatmaps were created with the ‘multiple variables’ feature in Prism. Welchs’s *t* test was used when two groups were compared, and one- or two-way ANOVA when there were more than two groups. No corrections were made for multiple comparisons, and data were assumed to be normally distributed. Statistical tests were two-sided. Data are expressed as mean ± SEM unless indicated otherwise in the figure legends. No samples, data points or animals were excluded from the final analyses. All replicates are biological, from different batches of SC-islets, mice or SC-islet grafts. PET imaging was conducted blinded but PET analysis was not blinded. Histological analyses were conducted using an automated, blinded-by-design pipeline.

## Results

### Study set-up and validation of tracer receptor expression in SC-islet grafts

**Fig. 1:**
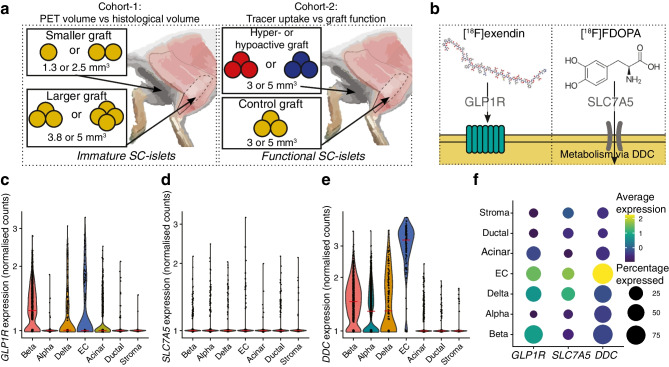
Study set-up and tracer cell type specificity. (**a**) Study set-up: cohort-1 consisted of comparison of different-sized SC-islet grafts in each leg; cohort-2 consisted of comparison of SC-islet grafts of different functional phenotypes in each leg. Immature SC-islets were differentiated as in the Balboa et al 2018 protocol [49], and functional SC-islets were differentiated as in the Barsby et al 2022 protocol [50]. Here, 1 mm^3^ corresponds to the volume of 566 IEQs (islets of 150 µm diameter) or 150 SC-islets (of 230 µm diameter). (**b**) PET tracers used in the study: [^18^F]exendin and [^18^F]FDOPA. (**c**–**f**) Tracer target expression in different cell types in SC-islet kidney subcapsular grafts at 6 months post implantation using single-cell RNA sequencing data from Balboa et al 2022 [2]. Red lines, median. The panels show *GLP1R* (**c**), *SLC7A5* (**d**) and *DDC* (**e**) mRNA expression in the different cell types, and the percentages of different cell types expressing the tracers (**f**)

We implanted human SC-islets into calf muscles of immunocompromised mice in two cohorts (Fig. 1a): the first with different volumes of SC-islets (cohort-1) and the second with control SC-islets or genetically engineered hyper- or hypoactive SC-islets (cohort-2). The Balboa et al 2018 differentiation protocol was used for cohort-1, and the Barsby et al 2022 protocol for cohort-2, reported to yield immature [49] or functionally primary islet-like SC-islets, respectively [2, 50]. We followed both cohorts for 5 months with PET imaging using two tracers: [^18^F]exendin and [^18^F]FDOPA (Fig. 1b). We chose the muscle site to avoid non-specific background from the bladder and kidneys, as both tracers are excreted in urine [46, 54].

To verify expression of the tracer receptors/carriers in SC-islets, we reanalysed single-cell RNA sequencing data from 6 month SC-islet grafts [2]. SC-islet grafts expressed *GLP1R* mostly in beta and delta cells as expected [41, 42, 55], but also in enterochromaffin-like cells (EC cells), a known endocrine impurity [2, 3] (Fig. 1c, ESM Fig. 1). All cell types expressed the amino acid transporter *SLC7A5* (a carrier for [^18^F]FDOPA) at low levels (Fig. 1d). All endocrine cell types, but especially the EC cells, expressed *DDC*, the rate limiting enzyme in DOPA metabolism (Fig. 1f).

### *KCNJ11*^*+/+*^, *KCNJ11*^−/−^ and *KCNJ11*^+/R201H^ SC-islets exhibit expected phenotypes in vitro

**Fig. 2:**
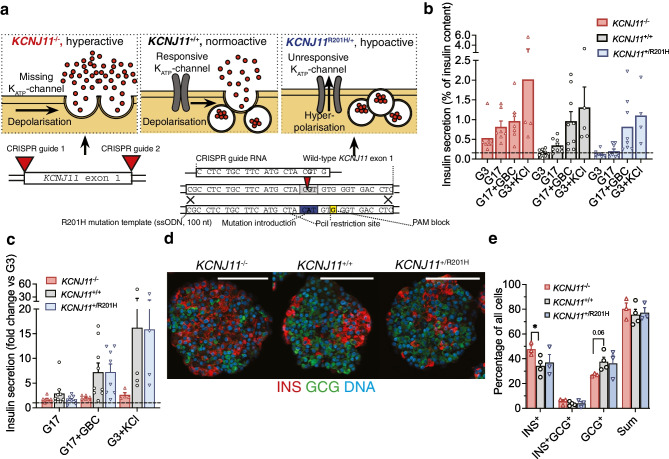
*KCNJ11* mutant SC-islet phenotypic characterisation in vitro. (**a**) Simplified molecular mechanism and genome editing strategy underlining the hyper-, normo- and hypoactive phenotypes of SC-islet beta cells in cohort-2. *KCNJ11*^−/−^ mutation leads to loss of K_ATP_-channel expression, leading to constant depolarisation and insulin secretion. *KCNJ11*^R201H/+^ mutation prevents K_ATP_-channel closure under high glucose, leading to inactive beta cells. nt, nucleotide; PAM, protospacer adjacent motif; ssODN, single-stranded oligonucleotide. (**b**) Percentage of total insulin content secreted in 2.8 mmol/l (G3) or 16.7 mmol/l (G17) glucose, or G17 and 100 nmol/l GBC, or G3 and 30 mmol/l KCl after 6 weeks of maturation culture. The horizontal line indicates the secretion rate in G3 in *KCNJ11*^+/+^ cells. (**c**) Same analysis as in (**b**) normalised against secretion in G3. (**d**, **e**) Immunohistochemistry (**d**) and quantification (**e**) of INS^+^ SC-beta cells and glucagon (GCG)^+^ SC-alpha cells at 6 weeks of maturation culture; scale bars, 100 µm. *p<0.05 by one-way ANOVA

To allow later examination of the consequences of stem cell-derived beta cell (SC-beta cell) functional status on tracer uptake, we sought to engineer SC-beta cells that are hyper- and hypoactive in their function by genome editing the K_ATP_-channel gene *KCNJ11*, critical for triggering insulin secretion [56]. We edited the *KCNJ11* locus to harbour a homozygous knockout of the gene (*KCNJ11*^−/−^), and, separately, introduced a gain-of-function mutation (*KCNJ11*^+/R201H^) (Fig. 2a, ESM Fig. 2). These mutations cause CHI and persistent neonatal diabetes, respectively. To validate the three phenotypes, we quantified insulin secretion responses in vitro to glucose and the sulfonylurea glibenclamide (GBC), a K_ATP_-channel agonist. *KCNJ11*^+/+^ SC-beta cells increased insulin secretion in response to high glucose, GBC and KCl as primary islets would [2]. *KCNJ11*^−/−^ SC-beta cells secreted three times more insulin in low glucose compared with *KCNJ11*^+/+^ SC-beta cells (Fig. 2b) and were unresponsive to GBC (Fig. 2c). Conversely, the *KCNJ11*^+/R201H^ SC-beta cells were unresponsive to glucose, but hyper-responsive to GBC (Fig. 2c). These SC-islets matched previously derived mature SC-islets in their cytoarchitecture and proportions of SC-beta cells and stem cell-derived alpha cells (SC-alpha cells) (Fig. 2d,e). The *KCNJ11*^*−/−*^ mutation slightly biased differentiation towards SC-beta cells at the expense of SC-alpha cells, as in our previous CHI model [48] (Fig. 2e). The SC-islets thus recapitulated the hyper- and hypoactive phenotypes.

### Engrafted SC-islets develop into either large impure grafts or small pure grafts

**Fig. 3:**
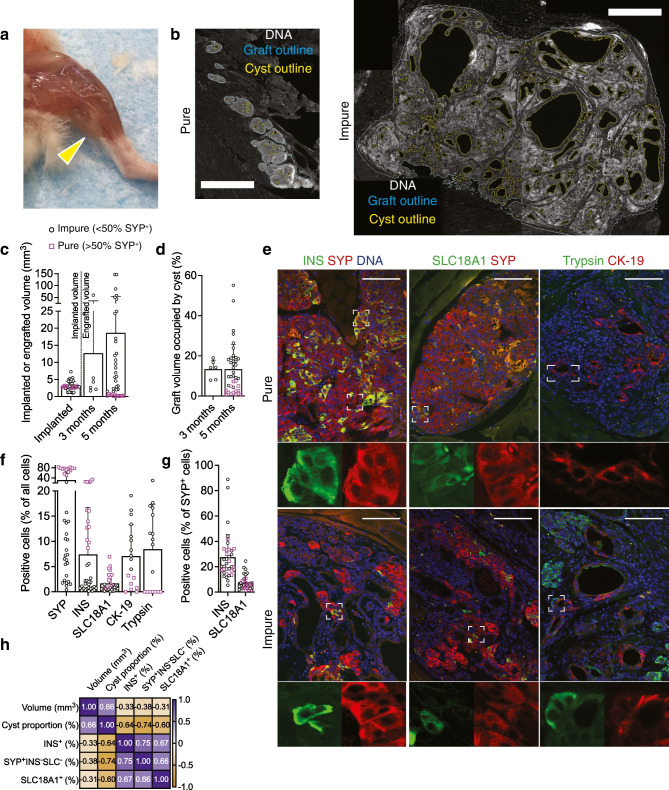
Characterisation of the SC-islet grafts. (**a**) Skinned mouse hind leg at 5 months post implantation; arrow indicates graft. (**b**) Hoechst-stained muscle graft sections used for volume determination; examples of pure and impure grafts in the same scale (bar, 1000 µm). Sections are displayed as CellProfiler pipeline output images highlighting the total (blue) and cyst-free (yellow) areas. (**c**) Implanted SC-islet volume and histologically determined graft volume. Pink squares, ‘pure’ with >50% SYP^+^ endocrine cells out of all graft cells; black circles, ‘impure’ with <50% SYP^+^ endocrine cells. (**d**) Cyst proportion of the grafts, quantified from images such as (**b**). (**e**) Immunohistochemistry analysis of SYP, the SC-beta cell marker INS, the EC cell marker SLC18A1, the acinar cell marker trypsin and the ductal cell marker CK-19; scale bar, 100 µm; insets display ×4 magnification. Examples are of pure and impure *KCNJ11*^+/+^ grafts. (**f**) Quantification of immunohistochemistry as percentage of all cells in the graft including connective tissue. (**g**) Percentage of INS^+^ and SLC18A1^+^ cells in SYP^+^ endocrine cells. (**h**) Pearson’s correlation of the composition features. The scale bar displays the spectrum of colour associated with the spectrum of correlation coefficients from −1 to 1. Data in (**c**), (**d**), (**f**) and (**g**) are displayed as mean ± SD

After the final 5 month imaging time point we euthanised the mice and retrieved the graft-bearing muscles. The grafts were sometimes visible as lightly coloured areas (Fig. 3a). We quantified the volume of the SC-islet grafts (*N*=36) by sectioning through the graft-bearing muscle and staining sections for graft area. Total and ‘cyst-free’ graft volumes were determined separately (Fig. 3b). The grafts consisted of either SC-islets with interspersed muscle fibres or a fused, heterogenous graft with cysts (Fig. 3b, ESM Fig. 3a). SC-islet graft volume was highly variable, ranging from <1 mm^3^ to 150 mm^3^, compared with implanted volume of 1–5 mm^3^ (Fig. 3c, ESM Fig. 3b).

Fluid-filled cysts and ductal mRNA expression are reported to be present in SC-islet grafts [2, 10, 12–15]. Cyst proportion was highly variable in the grafts, ranging from 1% to 50% of the total volume (Fig. 3d, ESM Fig. 3c). The contribution of synaptophysin-positive (SYP^+^) endocrine cells of all graft cells (including connective tissue) separated the grafts into two groups. Many grafts had ≈75% SYP^+^ cells (‘pure’ grafts), but the rest had <20% SYP^+^ cells (‘impure’ grafts) (Fig. 3e,f, ESM Fig. 3d). The pure grafts were derived with the Barsby et al 2022 protocol [50], but occasional impure grafts were generated as well.

The main tracer target cells, namely insulin (INS)^+^ SC-beta cells and solute carrier family 18 (vesicular monoamine transporter), member 1 (SLC18A1)^+^ EC cells were found in all grafts. SC-beta cells represented 10–30% of all cells (including connective tissue cells) in the pure grafts, but <5% in the impure grafts, while EC cells were present in all grafts at ≈1–5% of all cells (Fig. 3e,f, ESM Fig. 3d). Among endocrine cells, SC-beta cells contributed ≈30% and EC cells ≈10% (Fig. 3g, ESM Fig. 3e). Much of the non-endocrine tissue represented connective tissue, but we also analysed other pancreatic lineages, such as trypsin^+^ acinar cells and cytokeratin-19 (CK-19)^+^ ductal cells. No acinar cells were detected in the pure grafts, but they were present in the impure grafts (Fig. 3e,f, ESM Fig. 3d). The cysts were positive for CK-19, indicating the expected pancreatic ductal origin. Even the pure grafts that were devoid of organised cysts had interspersed CK-19^+^ cells representing ≈1% of all cells (Fig. 3f, ESM Fig. 3d). Large graft volume correlated with high cyst proportion and low number of endocrine cells (Fig. 3h). Pure *KCNJ11*^−/−^ grafts maintained a slightly higher SC-beta cell proportion among endocrine cells (ESM Fig. 3f) than *KCNJ11*^+/+^ grafts, similar to our previous study [48].

In summary, the grafts were highly variable in size, cyst proportion and purity. This allowed exploration of the relationship between graft composition and tracer uptake, with high clinical relevance regarding SC-islet graft safety and effectiveness.

### [^18^F]exendin and [^18^F]FDOPA PET accurately quantify graft size

**Fig. 4:**
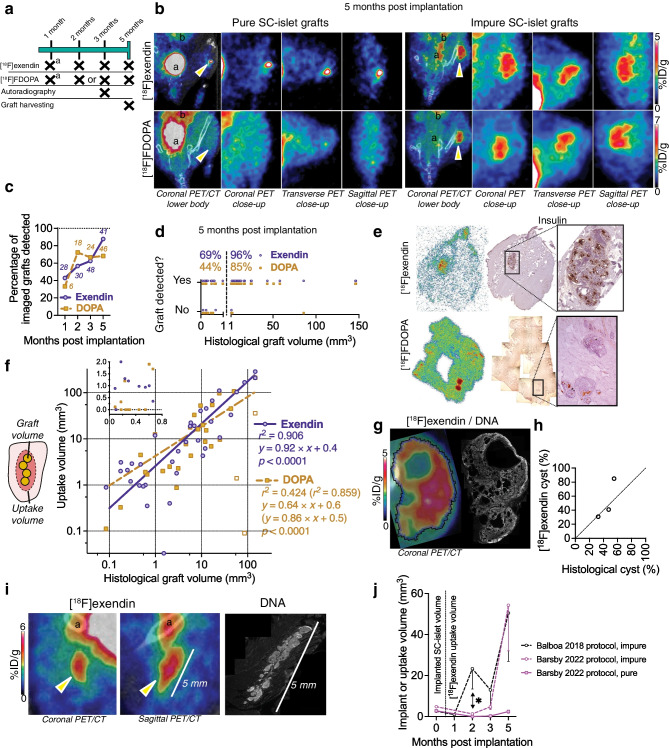
Follow-up and quantification of SC-islet graft volume with PET. (**a**) Imaging follow-up schedule; crosses indicate imaging, ex vivo autoradiography and harvesting time points. ^a^Cohort-1 only. (**b**) Views of PET/CT and PET images (summed 30 min scans); arrows indicate uptake volume considered the graft; ^a^bladder, ^b^kidneys. (**c**, **d**) Tracer detection performance per time point (*N* indicated on graph) (**c**) or according to actual graft volume (**d**) as a fraction of all imaged grafts detected. (**e**) Ex vivo autoradiography of muscle sections taken immediately after imaging with [^18^F]exendin or [^18^F]FDOPA and insulin immunohistochemistry from adjacent sections. (**f**) Linear regression between [^18^F]exendin and [^18^F]FDOPA uptake volume and the histologically determined graft volume (including cysts). Inset depicts low values. Values in parentheses are for [^18^F]FDOPA when three of the most cystic grafts are excluded (white-filled squares). (**g**) Example frame and VOI (blue) in [^18^F]exendin PET/CT highlighting low-uptake areas inside the VOI, and Hoechst-stained section of the same graft with corresponding cysts. (**h**) Cyst proportion determined by histology or PET (by drawing full and cyst-free uptake volumes). (**i**) Example of an elongated pattern in [^18^F]exendin PET/CT (^a^bladder) and a Hoechst-stained section of the same graft. (**j**) Graft size follow-up with [^18^F]exendin PET, grouped by final purity and SC-islet differentiation protocol, with grafts with >50% of all cells SYP^+^ considered pure. *n*: Balboa impure = 17, Barsby impure = 11, Barsby pure = 13. *p<0.05 by unpaired Welch’s *t* test

We followed the mice for 5 months with PET imaging, followed by histological analysis (Fig. 4a). Uptake of [^18^F]exendin and [^18^F]FDOPA was detected in vivo in calf muscles where the SC-islets were implanted (Fig. 4b), while the grafts were undetectable with CT alone. We drew the borders of the uptake volume using signal-to-background difference, which was slightly higher for [^18^F]exendin than for [^18^F]FDOPA (ESM Fig. 4a,b). Graft detection rate improved over time, reaching ≈90% for [^18^F]exendin and ≈70% for [^18^F]FDOPA by the 5 month time point (Fig. 4c). We then used the resolution of the PET scanner, ≈1 mm^3^, as a cut-off to calculate detection rate in relation to graft size. All but one graft >1 mm^3^ were detected with [^18^F]exendin (96% detection rate), with [^18^F]FDOPA also performing well (85% detection rate) (Fig. 4f). [^18^F]exendin even detected some grafts <1 mm^3^ (69% rate), suggesting very high [^18^F]exendin uptake in this small volume (Fig. 4d). Detection of grafts <1 mm^3^ was unreliable with [^18^F]FDOPA (44% rate) (Fig. 4d).

Both tracers reached a steady-state concentration within 5 min after injection, which remained on a high level for the 30 min scan, suggesting the uptake corresponded to tracer-targeted SC-islet tissue (ESM Fig. 4b). For further evidence of graft specificity, we euthanised a subset of mice immediately after imaging for ex vivo autoradiography. The autoradiography sections showed uptake of both tracers at locations staining positive for INS in adjacent sections (Fig. 4e), verifying that the uptake in graft-bearing muscle is graft specific.

Importantly, we then correlated uptake volume of both tracers with histologically determined graft volume. The uptake volume of [^18^F]exendin correlated very strongly (*r*^2^=0.91) with actual graft volume (Fig. 4f), indicating high accuracy for graft size quantification. [^18^F]FDOPA uptake volume also strongly correlated with actual graft volume when analysing all but the most cystic grafts (*r*^2^=0.86). However, the precise outlines of three highly cystic grafts were difficult to determine with [^18^F]FDOPA, reducing the strength of correlation when included (*r*^2^=0.42) (Fig. 4f). [^18^F]exendin readily visualised their outlines and the largest cysts inside (Fig. 4g, ESM Fig. 4c,d). By including or excluding the cysts in the uptake volume, we could non-invasively determine the cyst proportion, which closely resembled the histologically determined cyst proportion of these three grafts (Fig. 4h). The SC-islets were implanted while retracting the needle, dispersing the SC-islets. We could observe an elongated pearls-on-a-string engraftment pattern in the pure SC-islet grafts, over a distance of up to 5 mm, in histology. The corresponding PET scan revealed [^18^F]exendin uptake with the same dimensions (Fig. 4i), indicating grafts could be visualised despite lengthwise dispersion.

Finally, we analysed the longitudinal uptake patterns of the scanned SC-islet grafts. The grafts displayed either consistent expansion or stability, with tracer uptake concentration also remaining relatively constant during follow-up (ESM Fig. 4e,f). After grouping the grafts by final endocrine purity, the grafts remaining pure at 5 months (all derived with the Barsby et al 2022 protocol) were stable in longitudinal imaging (Fig. 4j). Interestingly, the impure grafts derived with the Balboa et al 2018 protocol and the occasional impure grafts derived with the Barsby et al 2022 protocol showed graft expansion at different times, at 2 or 5 months, respectively (Fig. 4j). These results demonstrate that the SC-islet protocol influences graft expansion, and that different growth patterns can be detected using PET.

Taken together, these data demonstrate the validity of [^18^F]exendin and [^18^F]DOPA PET for longitudinal monitoring and quantifying the actual SC-islet graft volume non-invasively across a wide distribution of graft volumes and purities.

### [^18^F]exendin uptake estimates graft SC-beta cell proportion and is unaffected by SC-beta cell functional state

**Fig. 5:**
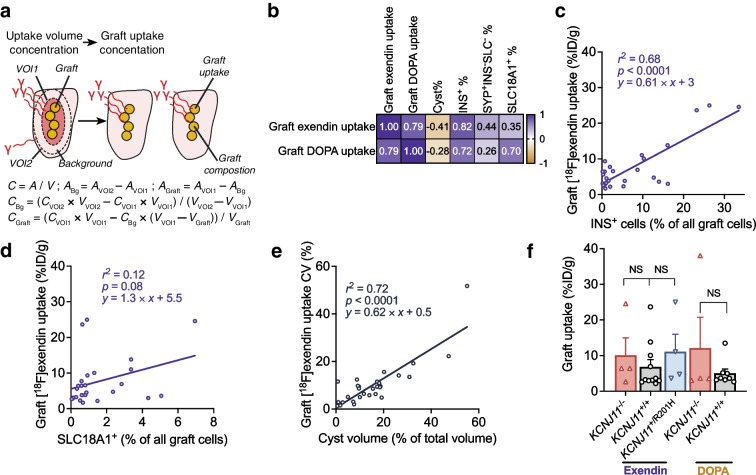
Correlation of graft composition and genotype with tracer uptake. (**a**) Schematic and associated formulas for volume dilution correction to determine graft uptake concentration. *C*, tracer uptake concentration; *A*, radioactivity (Bq); *V*, volume (mm^3^); Bg, background; VOI1, uptake volume; VOI2, additional VOI encompassing the whole muscle. (**b**) Pearson’s correlation of graft tracer uptake with different composition parameters. (**c**, **d**) Linear regression of graft exendin uptake and graft SC-beta cell (**c**) and EC cell (**d**) proportions. (**e**) Linear regression of graft [^18^F]exendin uptake variability and cyst proportion. CV = uptake SD/graft uptake mean × 100. (**f**) Graft [^18^F]exendin and [^18^F]FDOPA uptake in cohort-2 grafts of different genotypes. A single *KCNJ11*^+/R201H^ graft was detected with DOPA and is not plotted

We then investigated which aspects of graft composition and function determine uptake of the tracers; or, conversely, whether tracer uptake can be used to inform about compositional or functional aspects non-invasively. As described, the pure grafts were small, <1 mm^3^, but mostly still detectable due to high tracer uptake spilling over to expand the uptake volume. In this situation, much of the uptake volume consists of the muscle, diluting the measured graft uptake concentration. However, with background uptake measurements and histologically determined graft volume, we could calculate graft volume-specific uptake concentration, correcting for the dilution (Fig. 5a, Methods).

To explore which aspects of the graft composition determine tracer uptake, we correlated graft uptake concentration with histologically determined cyst proportion and cell type percentages from the same graft. Pearson’s correlation of tracer uptake and composition parameters showed negative correlation with cyst proportion and positive correlation with SC-beta and EC cell proportions (Fig. 5b). Analysed individually with linear regression, graft SC-beta cell percentage was positively correlated with both [^18^F]exendin (*r*^2^=0.68) and [^18^F]FDOPA (*r*^2^=0.52) uptake (Fig. 5c, ESM Fig. 5a). The percentage of EC cells was associated with higher uptake of [^18^F]FDOPA only (*r*^2^=0.48) (Fig. 5d, ESM Fig. 5b). Graft uptake of [^18^F]exendin and [^18^F]FDOPA did not correlate with the percentage of SYP^+^INS^−^SLC18A1^−^ cells (i.e. SC-alpha cells and stem cell-derived delta cells) (ESM Fig. 5c). As multiple factors contributed to the uptake, we analysed them simultaneously using a multiple regression model. The model had a better fit with graft [^18^F]exendin uptake (*R*^2^=0.81) than with any of the parameters alone, with percentage of SC-beta cells the most important determinant for [^18^F]exendin uptake (ESM Fig. 5d,e). As most of the pure and small grafts were undetected, analyses on [^18^F]FDOPA relied on fewer observations, and we state conclusions on its uptake determinants with less confidence. These data show that the real-world determinants of tracer uptake correspond well to the pattern predicted based on tracer target expression in single-cell RNA sequencing (Fig. 1c). Our observations also serve as proof-of-concept of the possibility of non-invasively informing about SC-islet graft SC-beta cell percentage.

In addition to the directly visualised cysts (Fig. 4i), uptake concentration in other impure grafts was highly variable, which could be translated into the CV of tracer uptake. This [^18^F]exendin uptake variation correlated with cyst proportion (Fig. 5e) (*r*^2^=0.72, using graft uptake for the calculations), suggesting cyst proportion could be estimated non-invasively even when individual cysts are too small to be visualised.

The cohort-2 mice carried a hyperactive *KCNJ11*^−/−^ or a hypoactive *KCNJ11*^+/R201H^ graft in one leg and a normoactive *KCNJ11*^+/+^ graft in the other. We next compared graft tracer uptake in these functionally different grafts and found no significant differences (Fig. 5f). Graft genotype had low influence on tracer uptake also in the multiple regression model (ESM Fig. 5d). Graft genotype did not affect the uptake dynamics of either tracer (ESM Fig. 5f,g). This indicates that both tracers can be confidently used in SC-islet grafts exhibiting various states of beta cell functionality as even the phenotypic extremes used here did not bias the tracer uptake. Taken together, composition of the graft, namely the proportions of SC-beta cells and EC cells, rather than functional status of its SC-beta cells, determines tracer uptake.

### Engrafted SC-islet graft volume and engrafted SC-beta cell mass poorly correlate with C-peptide measurements

Finally, to contextualise our findings with PET, we explored the ability of C-peptide measurements under fasted, glucose-stimulated and hypoglycaemic conditions to quantify graft size and beta cell mass. The imaged mice carried two grafts, making C-peptide measurements from them difficult to use. The sum of the grafts’ beta cell mass had no correlation with fasting C-peptide (ESM Fig. 6a). To better study the relationship between actual graft volume and C-peptide, we implanted an additional cohort of non-diabetic mice (*n*=14) with SC-islets under the kidney capsule in three doses, all coming from one batch of functional SC-islets.

**Fig. 6:**
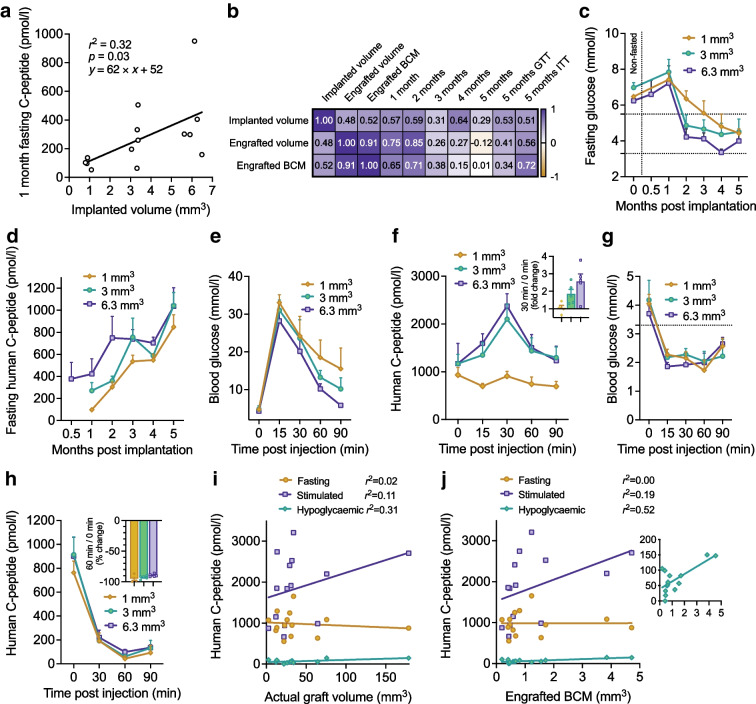
Relationship between C-peptide and implanted dose or engrafted volume. (**a**) Linear regression between SC-islet dose implanted under the kidney capsule and the fasted C-peptide level at 1 month post implantation. Implanted dose as total volume of SC-islets for each graft was quantified from stereomicroscope images. Here, 1 mm^3^ corresponds to 566 IEQs (150 µm diameter) or around 150 SC-islets (around 230 µm diameter). (**b**) Pearson’s correlation heatmap between implanted dose or histologically determined engrafted volume (total including cysts) or SC-beta cell mass (BCM) and C-peptide parameters. (**c**, **d**) Follow-up of fasting glucose (**c**) and fasting human-specific C-peptide (**d**) in the kidney cohort. *n*: 1 mm^3^ = 4; 3 mm^3^ = 5; 6.3 mm^3^ = 5. Low and high limits of human fasting normoglycaemia are indicated by horizontal lines. Vertical lines separate random-fed and fasted measurements. (**e**, **f**) Glucose (**e**) and C-peptide (**f**) following i.p. injection of glucose (3 mg/g) at 5 months post implantation, after a 5 h fast. (**g**, **h**) Glucose (**g**) and C-peptide (**h**) following i.p. injection of an insulin analogue (Actrapid, Novo Nordisk, Denmark) (0.75 mIU/g) at 5.5 months post implantation, after a 5 h fast. The low limit of human normoglycaemia is indicated by the horizontal line. (**i**, **j**) Linear regression of C-peptide parameters and total volume of the kidney graft (**i**) or the cyst-free, beta cell fraction-corrected graft volume (**j**). The C-peptide parameters were C-peptide in the fasted state (mean of 2 days) or 30 min after glucose injection or 60 min after insulin injection. The inset graph displays the same data with the *y*-axis scale focused to values below 200 pmol/l. Data in (**c**–**h**) are grouped by implanted SC-islet dose. Data in (**i**) and (**j**) display the histologically determined engrafted volume

One month after implantation, the correlation between implanted SC-islet dose and fasted C-peptide level was weak (Fig. 6a), and decreased in the subsequent 5 month follow-up (Fig. 6b). During the follow-up, the kidney grafts increased their fasting C-peptide secretion and lowered the mouse recipient’s fasting blood glucose level to human levels as expected [2], with minor differences between the doses (Fig. 6c,d). The C-peptide level was higher at 5 months in mice carrying a kidney graft compared with mice carrying two intramuscular grafts (ESM Fig. 6b). In a 5 month GTT, the mice with the lowest implanted dose failed to increase C-peptide secretion, while the mice that had received larger doses showed twofold responses (Fig. 6e,f). In a 5.5 month ITT, all grafts efficiently shut down their insulin secretion when the mice became hypoglycaemic, with ≈90% reduction from baseline (Fig. 6g,h). These data indicate the SC-islet grafts are functional in vivo, displaying glucose-regulated stimulation and shutdown of insulin secretion.

Following the ITT, we determined the actual engrafted SC-islet graft volume, cyst proportion and SC-beta cell proportion histologically (ESM Fig. 6c). Critically, the implanted SC-islet dose had low correlation with the actual engrafted SC-islet graft volume (Fig. 6b). We therefore used the actual graft volume for correlation against the various C-peptide measurements from the same mice: namely, the 5 month fasting level, peak C-peptide in the GTT and nadir C-peptide in the ITT. Even though the C-peptide measurements were human specific, none of these measurements correlated with the total engrafted SC-islet graft volume, which included cysts and non-beta cells (Fig. 6i). We then determined the graft SC-beta cell mass, by subtracting the cyst volume and multiplying the remainder with its SC-beta cell proportion. In linear regression, the SC-beta cell mass weakly correlated with the C-peptide secretion remaining under hypoglycaemia (*r*^2^=0.52), but not with fasting or stimulated C-peptide (Fig. 6j). These results from non-diabetic mice indicate that human-specific C-peptide measurements do not accurately quantify engrafted SC-islet volume including cysts, nor the engrafted SC-beta cell mass.

## Discussion

In this study, we demonstrate successful application of [^18^F]exendin and [^18^F]FDOPA PET as a robust SC-islet graft monitoring method. We show that the tracer uptake volume quantifies actual SC-islet graft volume, whether pure or impure. Additionally, characteristics of [^18^F]exendin uptake yield information about SC-islet graft SC-beta cell and cyst composition. This method enables study of SC-islet graft size and composition on a level not possible with any other non-invasive method.

We imaged the SC-islet grafts with [^18^F]exendin and [^18^F]FDOPA, providing perspective on their strengths and weaknesses (Table 1). Both performed well in quantifying graft size, but [^18^F]exendin was more sensitive than [^18^F]FDOPA in detecting the grafts, especially the small and pure ones. This heightened sensitivity was reflected in the ability of [^18^F]exendin to delineate large cysts inside the grafts. [^18^F]exendin uptake was predominantly determined by the proportion of SC-beta cells in the graft, while [^18^F]FDOPA uptake appeared to be determined by the proportion of both SC-beta and EC cells. The uptake of neither tracer was affected by extremes in beta cell insulin secretion functionality, which likely varies less extremely in different wild-type SC-islet batches. [^18^F]FDOPA has an important practical advantage, being readily available in PET centres for use in central nervous system and focal CHI imaging [44], while ^68^Ga- or ^18^F-labelled exendin is still limited to experimental use in dedicated centres.

**Table 1:** Comparison between [^18^F]exendin and [^18^F]FDOPA imaging

Parameter	[^18^F]exendin	[^18^F]FDOPA
Detection of grafts <1 mm^3^	++	+
Detection of grafts >1 mm^3^	+++	++
Quantification of graft volume	+++	++
Assessment of cyst proportion	+	0
Influence of SC-beta cell percentage on tracer uptake	++	+ (?)
Influence of EC cell percentage on tracer uptake	0	+ (?)
Influence of SC-beta cell functional state on tracer uptake	0	0
Clinical availability	0	++

Authors’ assessment of performance in the SC-islet graft context: +++, strong ability/effect; ++, moderate ability/effect; +, marginal ability/effect; 0, no ability/effect; (?), lower level of confidence

In recent years, multiple other tracers have been used for imaging islets in the native pancreas and after transplantation. These include [^11^C]HTP [38], which, similar to DOPA, is metabolised by DOPA decarboxylase (DDC); vesicular monoamine transporter type 2 (VMAT2)-targeting tracers [57]; and, more recently, prostaglandin D2 receptor 2 (GPR44) [58], dipeptidyl peptidase like 6 (DPP6) [59] and the DGCR2-targeting tracers [47]. All of these have slightly different binding profiles among endocrine cells, which might be leveraged depending on the application. In the SC-islet implantation and primary islet transplantation context, specificity over the acinar and ductal cells is of lesser concern than in the native pancreas. The newer tracers may have even higher uptake and sensitivity in SC-islets if their targets are expressed at a higher level than the GLP1R.

[^18^F]FDOPA visualises focal CHI lesions for an unknown reason. Hypothetically, it may be due either to hyperfunctionality of the lesion beta cells increasing their demand for metabolites such as DOPA or to the local increase in beta cells in the lesion. Based on our data showing that CHI grafts have similar [^18^F]FDOPA uptake as healthy and diabetic grafts, lesion visualisation is probably due to the locally increased number of beta cells. Pointing to the same conclusion, Boss et al found that focal CHI lesions can be better located with exendin PET than with DOPA PET [60], which, according to our data, would not be due to higher exendin uptake in hyperactive grafts. Even though beta cell functionality does not influence exendin uptake, extreme hyperglycaemia can increase it by increasing GLP1R expression [61].

The purity differences in the SC-islet grafts strengthened the conclusions of our imaging data but pose a challenge for clinical translation, as they have been reported by other groups as well. Rigorous pre-implantation quality control for the contaminating cell populations, such as lingering progenitors and non-pancreatic contaminants [14, 15], is needed as functional maturity does not necessarily equate to a risk-free graft. All the impure grafts from SC-islets differentiated with the Barsby et al 2022 protocol arose from two out of eight distinct pools of SC-islets used to prepare the grafts in cohort-2, with all the grafts from those two pools being impure. This suggests that the occurrence of impure grafts is not random, but is most likely due to unknown off-target cells in those pools. These two pools were combined from SC-islet cultures quality controlled for on-target cells with SYP flow cytometry, which reported 80–85% SYP^+^ endocrine cells (not shown), which was deemed sufficiently high, as quality control cut-offs have not been established in published works. Multiple non-endocrine off-target cell populations have been identified in SC-islets or SC-islet grafts, including SRY-box transcription factor 9 (SOX9)^+^chromogranin A^–^ non-endocrine progenitors [62]; pancreatic ductal and acinar cells; *FGB*-, *AGR2*- and *CDX2*-expressing liver and intestinal cells [14, 15]; *FOXJ2*-expressing cells [13]; vimentin^+^ mesenchymal cells [15]; and nestin^+^ neuronal and retinoid isomerohydrolase (RPE65)^+^ retinal pigment epithelium cells [15]. As our PET methods rely on detecting the on-target cells, a high number of off-target cells cannot be directly inferred from low signal. However, devising a tracer suitable for all off-target populations might be difficult.

The large variability in graft size was probably the result of two stochastic processes: initial cell loss on engraftment [29, 63] into muscle, a challenging implantation site [28]; and subsequent growth of the engrafted cells. Both are related to cell maturity, as more mature beta cells are more susceptible to hypoxia [29] and have lower proliferation capacity [64]. Large graft size, which was easily visualised with these methods, was strongly associated with impurity. These methods could thus be useful in supplementing pre-implantation quality control by informing decisions to intervene if the graft becomes too large or too impure.

In our study, all SC-islet doses resulted in similar C-peptide levels after sufficient time to mature in mice. Despite all mice being implanted with the same batch of SC-islets, those implanted with the lowest dose failed to increase C-peptide secretion on glucose injection. This mirrors another SC-islet implantation study, where a low dose of SC-islets in an animal model of diabetes failed to reverse diabetes, and, in subsequent glucose tolerance testing, failed to respond to the glucose stimulus [28]. C-peptide level has variably been reported to be either independent or positively correlated with transplanted primary human islet dose in mice [28, 65, 66] and human recipients [67, 68], probably owing to differences in islet quality, engraftment efficiency and how long after transplantation the C-peptide measurements were taken. However, correlations with the actual engrafted islet volume are lacking. We made these correlations and found that total engrafted graft size, which includes non-beta cells, does not correlate with any of the C-peptide measures. The engrafted SC-beta cell mass did not linearly correlate with fasted or glucose-stimulated C-peptide measurements. This is understandable as excess of beta cells should not lead to excessive insulin secretion if the SC-beta cells are physiologically regulated. On induced hypoglycaemia, the grafts efficiently shut down insulin secretion by 90% from fasted levels, indicating effective glucose sensing. Despite this, we could detect low levels of inappropriate insulin release. Interestingly, this insulin leak was a weak indicator of engrafted SC-beta cell mass.

A clear advantage of PET over methods such as bioluminescence imaging is its direct translatability to clinical use. In our study, the graft volume in many of the grafts was at the lower limit of the PET scanner’s spatial resolution (of ≈1 mm^3^), but the grafts could still be detected. Importantly, the larger human scale would likely greatly improve the ability of these tracers to quantify both graft size and composition, as the human scanners operate on approximately threefold lower resolution, but the graft size would need to be a thousandfold larger for therapeutic effectiveness [1]. The larger graft volumes would result in lower dilution in uptake concentration, resulting in more accurate quantification of graft composition, at least if the graft is not dispersed over a relatively larger area in humans. We showed that lengthwise dispersion of the grafts does not prevent SC-graft visualisation. The encapsulation devices used in certain SC-islet implantation trials (NCT03163511, NCT05791201) are likely visible in CT and MRI. CT/MRI quantification of the encapsulated graft volume could be followed by PET to quantify graft SC-beta cell composition accurately, as mathematical corrections for graft uptake similar to those using the histologically determined graft size could be made. This might be useful particularly in the encapsulation device context, as the explanted devices have shown a low SC-beta cell percentage [69, 70]. Extrapolating from biodistribution in rats, ^18^F-labelled exendin has a low enough radiation dose to justify repeated scans [54]. Altogether, it would probably be more feasible to visualise SC-islet grafts in humans than in mice. If these methods were translated, an SC-islet graft recipient imaged with either tracer could have the size of their graft quantified by the uptake volume. If a large uptake concentration was measured, a higher number of SC-beta cells could be inferred to be present in the graft, at least when using [^18^F]exendin.

One limitation to translating these findings is standard use of the intraportal site, also used in one of the ongoing SC-islet implantation trials (NCT04786262). Imaging intrahepatic islets is challenging, as they stochastically engraft next to the blood vessels, leading to signal diffusion and background. However, in a promising outcome for applying PET for intrahepatic SC-islet grafts, a recent study showed that primary islet grafts can be detected in the liver [37] with a ^68^Ga-exendin tracer. Additionally, while our method was highly applicable for long-term monitoring, it was limited in mapping early post-implantation events, as 60% of the imaged grafts were not detected at the 1 month time point. The reason for this remains unknown. While the SC-islets express the tracer receptors in vitro before implantation and exhibit strong responses to GLP1R agonists [2], they might lose some of the expression during the shocking early engraftment period. Lack of detection could also be related to incomplete development of vasculature. A similar PET study using primary rodent islets suggested that vasculature is sufficiently established by 3 weeks [71], but the SC-islets could have weaker vascularisation as they lack the endothelial cells found inside primary islets. Nevertheless, these early events could be visualised by pre-labelling the SC-islet material with superparamagnetic iron oxide particles for MRI [72, 73] or with [^18^F]FDG for PET [74], or, in a preclinical setting, with a bioluminescent reporter system [29, 75, 76]. Alternatively, a blood marker for beta cell death, such as cell-free insulin DNA with a beta cell-specific methylation pattern, could be used as an indirect measure of engraftment [77].

One limitation of the study design was that the relationship of SC-beta cell mass and C-peptide was explored in a cohort that was not imaged and that was implanted at a different site, preventing direct comparison of PET and C-peptide measurements. Neither the intramuscular- nor the subcapsular graft-carrying mice were rendered diabetic, preventing assessment of the therapeutic potential of the SC-islet grafts. Preservation of the mouse beta cells could contribute to the glucose clearing in the glucose tolerance testing, lowering the need for the human SC-islet grafts to secrete insulin. The intact mouse beta cells could theoretically influence correlations between human SC-islet graft volume and the human C-peptide, particularly in the glucose-stimulated conditions. Several SC-islet cultures were pooled as implantation batches to increase consistency in PET; however, this limits the ability to trace back origins of off-target growth to specific features of the pre-implantation cultures. Non-blinded PET data analysis was a limitation in study execution.

The strengths of this study include the comparison of two different tracers; inclusion and quantification of grafts with different compositions; validation of tracers in SC-islets with hyper- and hypofunctional SC-beta cells; and, to a lesser extent, the context given by the parallel assessment of C-peptide measurements.

In conclusion, we propose using [^18^F]exendin and/or [^18^F]FDOPA PET imaging as a method for longitudinal monitoring of SC-islet grafts. This would allow accurate quantification of graft size and estimation of aspects of graft composition, such as cyst and SC-beta cell proportion. PET imaging could help improve the safety and efficacy of SC-islet grafts as an emerging cell replacement therapy for insulin-deficient diabetes.

## Supplementary Information

Below is the link to the electronic supplementary material.

ESM (PDF 13534 KB)

## Data Availability

All data are available on reasonable request.

## Abbreviations

CHI: Congenital hyperinsulinism
[^11^C]HTP: [^11^C]-5-hydroxy-tryptophan
CK-19: Cytokeratin-19
CT: Computed tomography
DGCR2: DiGeorge syndrome critical region gene 2
DICOM: Digital Imaging and Communications in Medicine
DOPA: 3,4-dihydroxyphenylalanine
EC cell: Enterochromaffin-like cell
[^18^F]exendin: [^18^F]F-dibenzocyclooctyne-exendin-4
[^18^F]FDOPA: 6-[^18^F]fluoro-l-3,4-dihydroxyphenylalanine
GBC: Glibenclamide
GLP1R: Glucagon-like peptide 1 receptor
%ID/g: Percentage of injected dose per gram of tissue
IEQ: Islet equivalent
INS: Insulin
PET: Positron emission tomography
SC-alpha cell: Stem cell-derived alpha cell
SC-beta cell: Stem cell-derived beta cell
SC-islet: Stem cell-derived islet
SLC18A1: Solute carrier family 18 (vesicular monoamine transporter), member 1
SYP: Synaptophysin
VOI: Volume of interest
VOI1: VOI encompassing the putative graft
VOI2: VOI encompassing the calf muscle
