# Supplementary material for: Non-invasive quantification of stem cell-derived islet graft size and composition
Source: Diabetologia. 2024 Jun 14;67(9):1912–29. doi: 10.1007/s00125-024-06194-5 (PMC11410899; doi:10.1007/s00125-024-06194-5)
Supplement: Supplementary file 1 — ESM (PDF 13534 KB) [file 125_2024_6194_MOESM1_ESM.pdf]

ELECTRONIC SUPPLEMENTARY MATERIAL (ESM) FOR

Non-invasive quantification of stem cell derived islet graft size and composition

V. Lithovius et al.

This document includes:

Supplementary materials and methods:

- Genome editing of the *KCNJ11* locus
- Animal husbandry and C-peptide measurements
- Histological examination of graft volume and cyst proportion
- Histological examination of graft composition

ESM figures 1 to 6, with embedded captions

Genome editing of the *KCNJ11* locus

The H1 line was separately genome-edited to generate *KCNJ11* knockout and *KCNJ11* R201H-knock-in. Genome editing was performed using ribonucleoprotein (RNP) CRISPR-Cas9 system (Integrated DNA Technologies, USA), with guides and a mutation template designed using Benchling (Biology Software, 2017, USA). Two million cells were electroporated with 10 µg Alt-R™ S.p. Cas9 Nuclease V3 combined with gRNA and 4 µg of the 100-b ssODN mutation template (Integrated DNA Technologies, USA), using Neon Transfection system (Thermo Fisher; 1100 V; 20 ms; two pulses). To knockout *KCNJ11*, we targeted a 1.27 kb deletion spanning the protein-coding exon using 2 gRNAs (KO gRNA1: AGGCCCTAGGCCACGTCCGA and KO gRNA2: GTGTGTACACACGGACCATG). The deletion was confirmed with PCR using the following primers, *KCNJ11* KO Fw: CTCAGCCTCCCAACGTACTG and *KCNJ11* KO Rv: AGAGTGTGGCTGGTCAATCG. For knocking in the *KCNJ11*-R201H gain-of-function mutation, we used KI gRNA: CCTCTGCTTCATGCTACGTG and a mutation template harbouring a silent mutation to create a PciI restriction site facilitating screening for edited clones. Monoclonal cells were isolated using limiting dilution, expanded, and screened using PCR and Sanger sequencing, with the following primers used for screening: *KCNJ11* KI Fw: TCATCGTGCAGAACATCGTG and *KCNJ11* KI Rv: ATCTCATCGGCCAGGTAGGA. One homozygous knockout and two heterozygous R201H knock-in clones were produced. The top three CRISPR off-target hits for each gRNA were sequenced demonstrating no insertions or deletions. G-band karyotyping was performed at Ambar Lab, Barcelona, Spain.

Animal husbandry and C-peptide measurements

After implantation, the mice were given analgesics for two days and first housed for two weeks in Helsinki and then transported to the University of Turku animal facility for 5-month PET imaging follow-up. The kidney cohort used for C-peptide studies was kept in the University of Helsinki animal facility. Both were housed in standard conditions in Scantiner or individually ventilated cage system, fed irradiated standard chow *ad-libitum*, and kept on a 12h dark/12h light cycle. They were monitored for well-being by animal facility staff and the researchers. C-peptide measurements were taken from *vena saphena magna* or via cardiac puncture. Glucose- and insulin tolerance tests were conducted in the afternoon after a 5-hour fast by injecting 3 mg/g of glucose or 0.75 mIU/g of insulin analogue (Actrapid, Novo Nordisk, Denmark) intraperitoneally, with blood sampling intervals as indicated in the figures. Saline injections were given after blood sampling. Sample C-peptide concentration was analysed with a human

specific ultrasensitive ELISA kit (10-1141-01, Mercodia, Sweden), as per manufacturer instructions. The animal care and experiments were approved by the Finnish national animal experiment board (ESAVI/14852/2018, ESAVI/9734/2021, ESAVI/12143/2019 and ESAVI/16273/2022).

Histological examination of graft volume and cyst proportion

After the final 5-month imaging timepoint, the mice were sacrificed and their calf muscles harvested whole with a scalpel, fixed for 48h in 4% PFA in +25C, followed by paraffin-embedding. These graft-bearing muscles were sectioned through while recording the graft depth between the first and last graft-containing section. Along the depth, at least 8 sections were stained with Hoechst separating nuclei-rich grafts and nuclei-sparse muscle; or analysed unstained based on separation between green autofluorescence-high muscle from autofluorescence-low grafts. The section graft area was imaged (EVOS, Thermo Fisher) with 4x magnification and the images stitched using the "pairwise stitching" algorithm on Fiji *[1]*. These graft area on these stitched images was calculated on a custom CellProfiler (version 4.0) *[2]* pipeline, by drawing the graft outlines followed by two-time thresholding of the area to include or exclude cysts, quantifying total and "cyst-free" graft areas in mm^2. The total and cyst-free graft volumes were then calculated using the depth and the total and cyst-free areas.

Histological examination of graft composition

Immunohistochemistry was conducted by running a standard deparaffinization series, followed by 30-minute HIER in +95C in sodium citrate buffer (pH 6), followed by 10-minute blocking (UltraV block, Thermo Fisher, TA-125-UB) and overnight application of primary antibodies in +4C. Secondary antibodies were applied for 45 minutes in +25C and slides mounted with ProLong antifade mountant (Thermo Fisher #P36984). The following primary antibodies were used: gp-insulin (Agilent/DAKO IR002, 1:2), mo-glucagon (Sigma-Aldrich #G2654, 1:500), mo-synaptophysin (Agilent/DAKO M7315, 1:200), rb-SLC18A1 (Sigma-Aldrich #HPA063797, 1:200), pan-specific sh-trypsin (R&D systems, #AF3586, 1:100), rb-cytokeratin-19 (Abcam 15463, 1:200). The following secondary antibodies were used, all with 1:500 dilution from Thermo Fisher: anti-mouse red (#21203), anti-mouse green (#21202), anti-rabbit green (#21206), anti-rabbit red (#21207), anti-guineapig red (#11076), anti-guineapig green (#11073) and anti-sheep green (#11015). Slides were imaged on Zeiss AxioImager equipped with Apotome II. Graft composition was analysed from >10 random fields from 1-2 sections with a custom CellProfiler 4.0 pipeline, similar to one previously reported by our

group *[3]*. All images were analysed so that all non-muscle areas were considered part of the graft

ESM references

1. Preibisch S, Saalfeld S, Tomancak P (2009) Globally optimal stitching of tiled 3D microscopic image acquisitions. Bioinformatics 25(11):1463–1465. https://doi.org/10.1093/bioinformatics/btp184

2. Stirling DR, Swain-Bowden MJ, Lucas AM, Carpenter AE, Cimini BA, Goodman A (2021) CellProfiler 4: improvements in speed, utility and usability. BMC Bioinformatics 22(1):433. https://doi.org/10.1186/s12859-021-04344-9

3. Lithovius V, Saarimäki-Vire J, Balboa D, et al (2021) SUR1-mutant iPS cell-derived islets recapitulate the pathophysiology of congenital hyperinsulinism. Diabetologia 64(3):630–640. https://doi.org/10.1007/s00125-020-05346-7

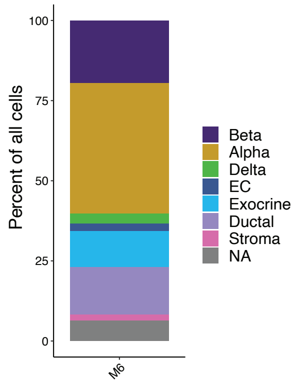

ESM Figure 1, cell type contributions to the single cell RNA sequencing:

Kidey subcapsular SC-islet grafts at 6 months post-implantation, re-analysed from *Balboa, Barsby & Lithovius et al. 2022 Nature Biotechnology* -data. EC = enterochromaffin -like cells

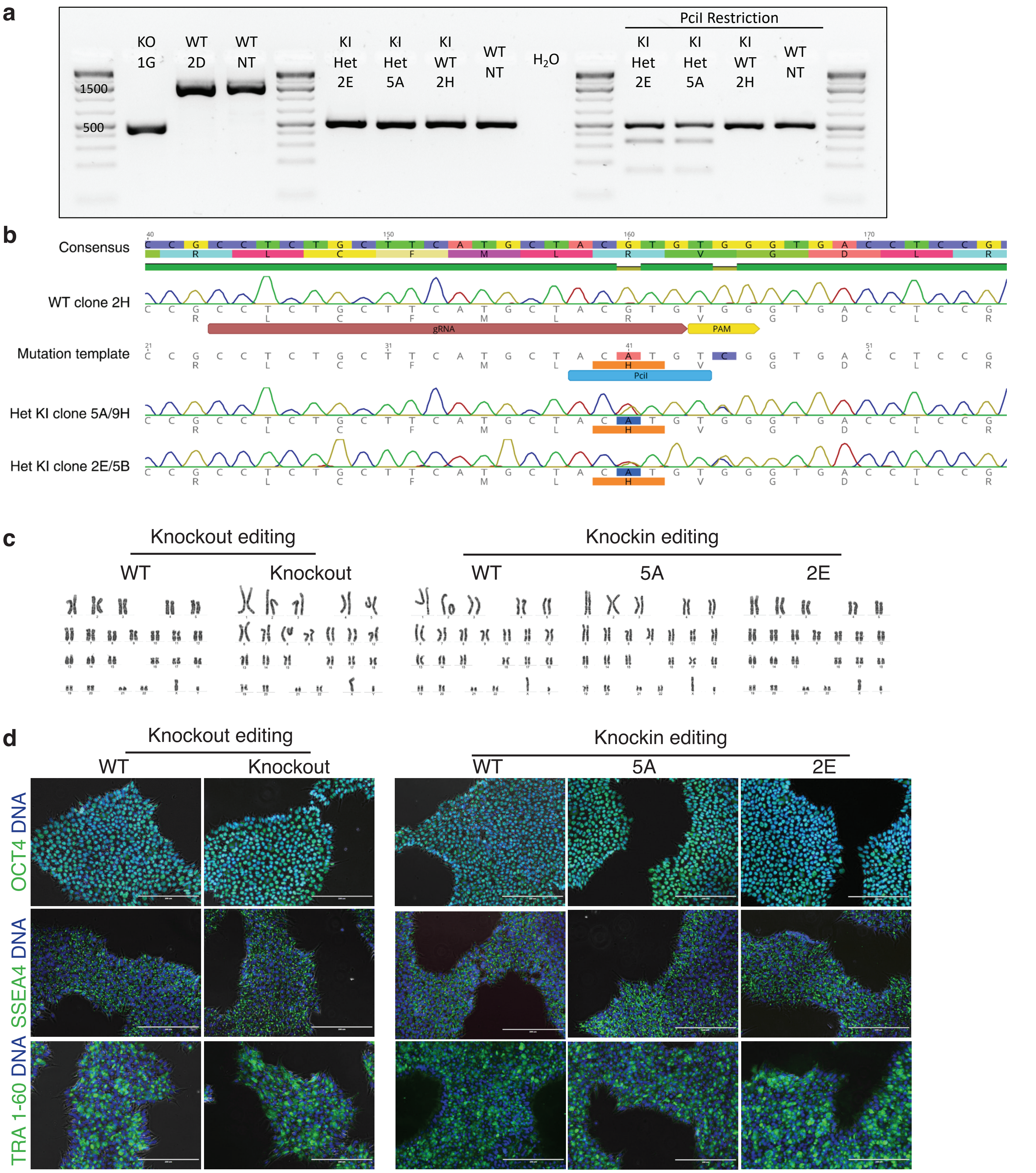

ESM Figure 2, quality control of *KCNJ11* knockout and R201H knockin editing:

a) PCR of the *KCNJ11* locus of the knockouts (left) and of the knocked-in area containing PciI restriction site (right) **b**) Sanger sequencing of the knockin clones **c**) G-band karyotyping of the knockout and knockin clones **d**) Immunohistochemistry for pluripotency markers OCT4 (Santa Cruz, sc-9081, 1:250), SSEA4 (Thermo Fisher, MA1-021-D488, 1:100) and TRA 1-60 (Thermo Fisher, MA1-023, 1:100) in the knockout and knockin clones

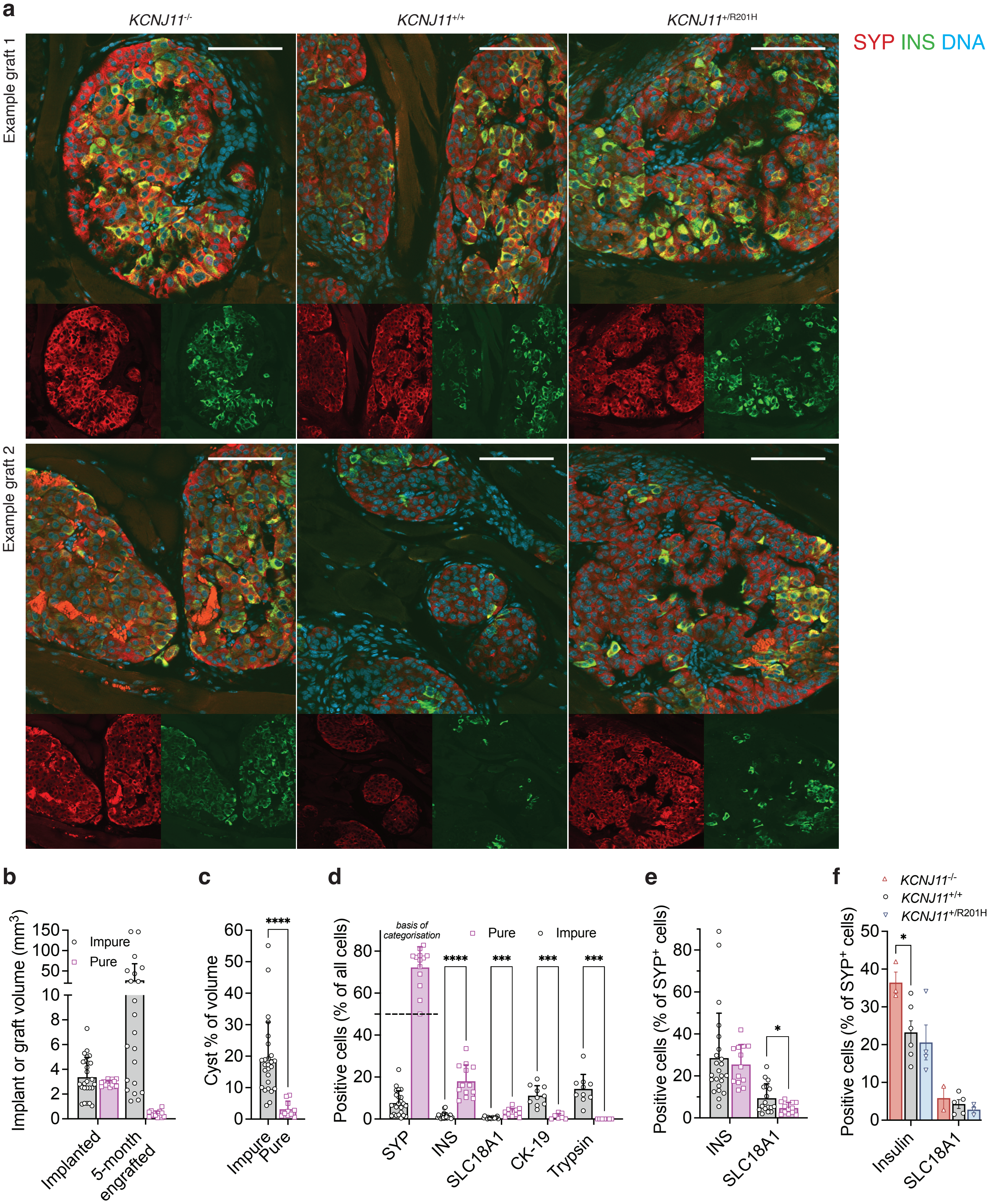

ESM Figure 3, composition data displayed by purity category and genotype:

a) Example immunohistochemistry of pure 5-month SC-islet grafts of different genotypes, stained for endocrine marker synaptophysin (SYP), SC-beta cell marker insulin (INS) and nuclear marker hoechst, scale bar 100 μm **b**) Implanted SC-islet volume and actual graft volume (including cysts) at 5-months post-implantation **c**) Cyst proportion of 5-month grafts **d**) Quantifications of immunohistochemisty, percentage of SYP⁺ served as basis for categorisation to “pure” and “impure”, cutoff at 50%. Enterochromaffin cell marker SLC18A1, acinar cell marker trypsin and ductal cell marker cytokeratin-19 (CK-19) **e-f**) Quantification of INS⁺ and SLC18A1⁺ populations as percentage of SYP⁺ endocrine cells, grouped by purity e) and genotype f). All display mean ±SD, One-way ANOVA or Welch’s t-test.

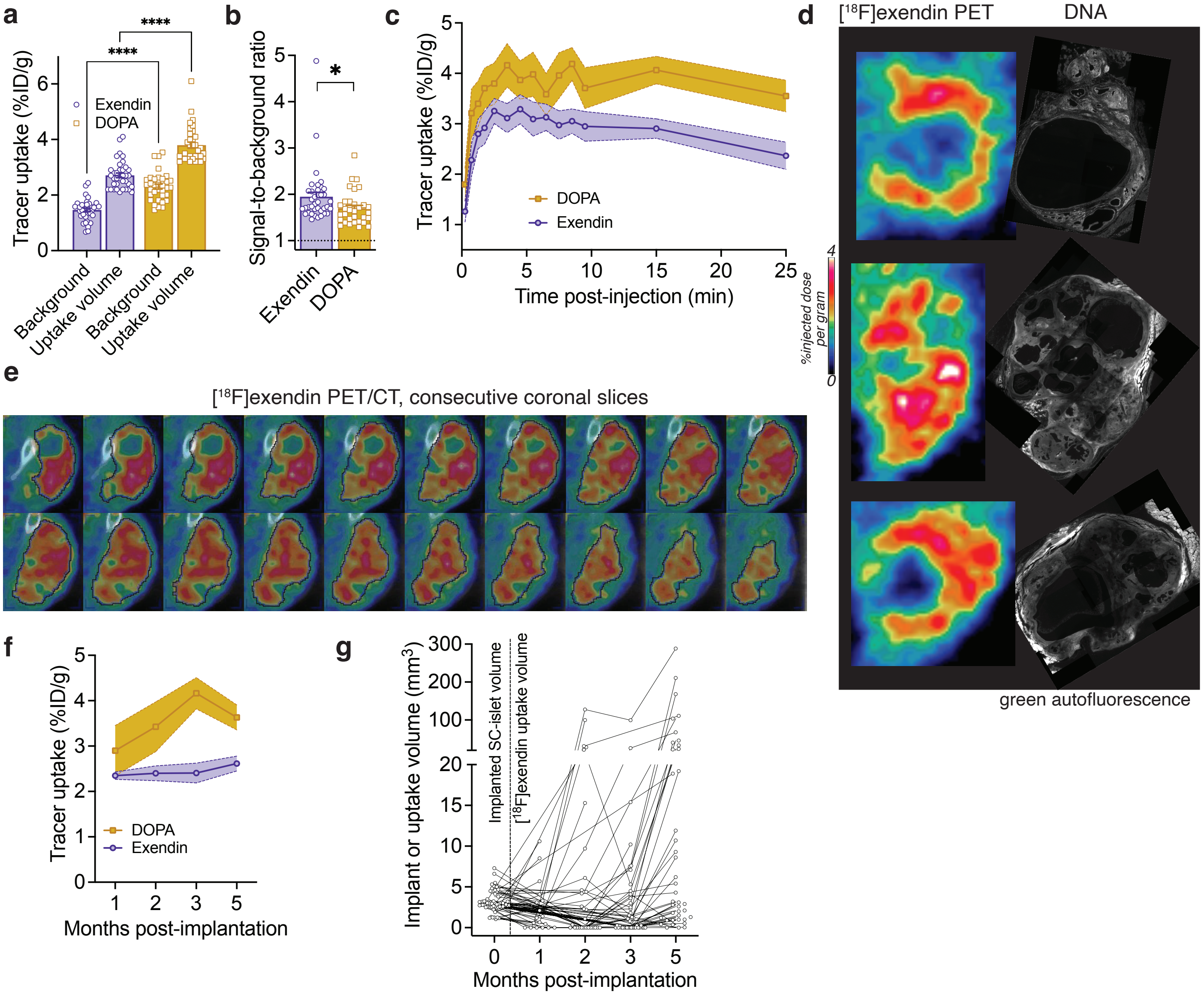

ESM Figure 4, additional PET data:

a) Absolute levels of tracer uptake at 5 months post-implantation in the uptake volume and in the background (surrounding muscle). Mean±SEM, Two-way ANOVA **b**) Ratio of tracer uptake concentration inside the uptake volume and the background. Mean±SEM, Welch's *t*-test **c**) Dynamic uptake of the tracers during imaging at 5 months post-implantation as percentage of injected tracer dose per gram (=ml) of graft volume. Mean ±95%CI, *N:* [^{18}F]exendin=36, [^{18}F]FDOPA=30, 25 min timepoint includes uptake from 20 to 30 minutes. **d-e**) Three individual SC-islet grafts displaying low uptake areas inside the uptake volume corresponding to cysts in histology (top hoechst, middle and bottom green autofluorescence) d). An additional graft where the uptake volume considered foreground (including cysts) is highlighted in 20 consecutive slices in PET/CT e), both at 5 months post-implantation **f**) Progression of uptake concentration of both tracers during follow-up. Mean ±95%CI **g**) Graft size follow-up with [^{18}F]exendin PET in individual grafts. Non-detected grafts marked with 0.

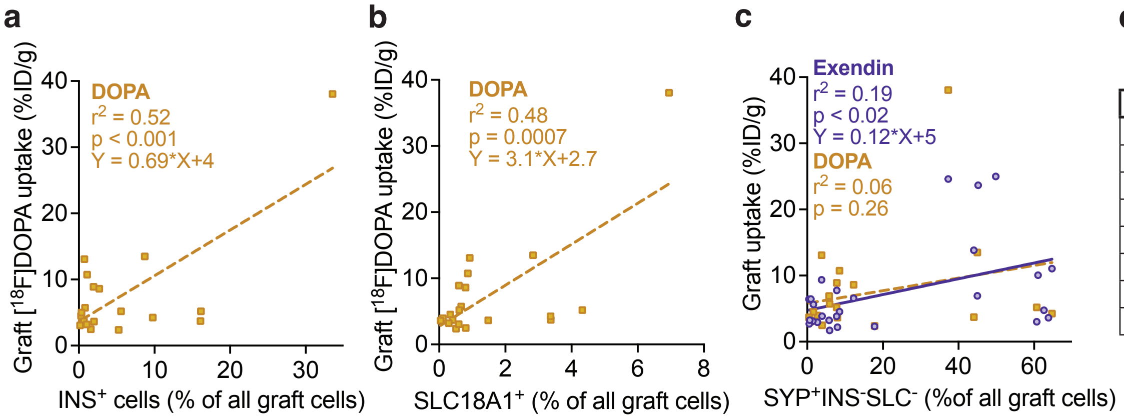

d

Variable	Estimate	SE	*p*
Intercept	4.72	2.4	0.07
Cyst %	-0.03	0.1	0.75
INS⁺ %	0.91	0.1	0.0001***
SYP⁺ %	-0.06	0.1	0.46
SLC⁺ %	-1.55	0.6	0.02 *
-/-	1.52	2.2	0.51
+/+	-0.02	2.2	0.99
R201H	-0.71	3.5	0.88

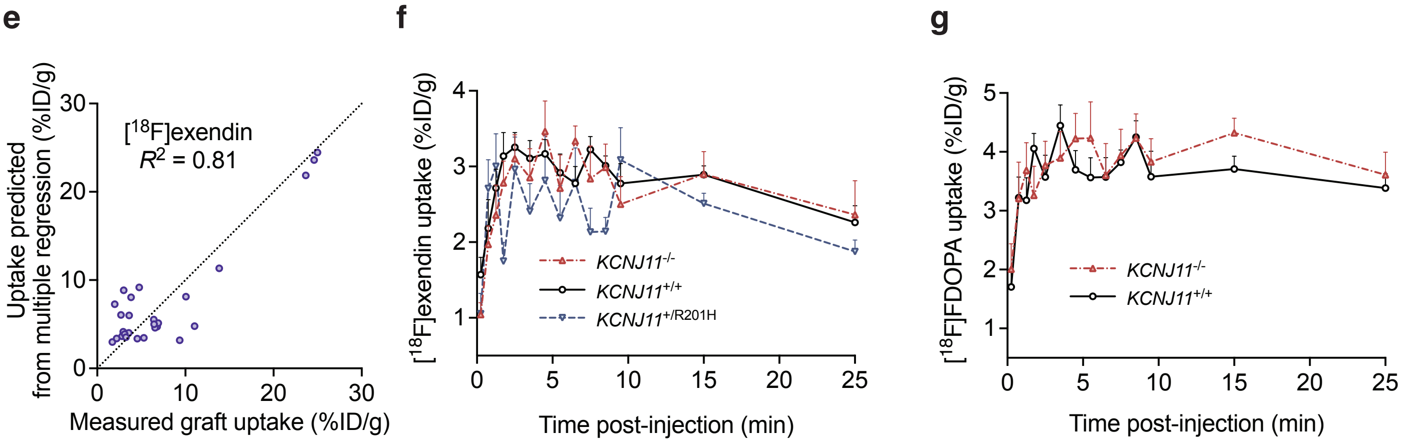

ESM Figure 5, Correlations of graft composition and genotype with tracer uptake:
a-c) Linear regression of graft uptake concentration and graft composition parameters: a) percentage of INS⁺ beta cells b) percentage of SLC18A1⁺ EC cell c) percentage of SYP⁺INS⁻SLC18A1⁻ endocrine cells (i.e. alpha and delta cells) of all graft cells **d-e**) Multiple linear regression of tracer uptake from composition, in the form: Y (Predicted uptake) = $\beta_{intercept}+\beta_{Cyst}*X_{Cyst}+\beta_{insulin}*X_{insulin}$... (X values are histological measurements from individual grafts). d) Model estimates of β, SYP⁺ = SYP⁺INS⁻SLC18A1⁻, SLC⁺ = SLC18A1⁺, -/-, +/+ and R201H refer to *KCNJ11* genotype. e) Correspondence of model values to measured graft uptake values. Model goodness-of-fit R^2 and line-of-identity displayed. **f-g**) Dynamic uptake of [^{18}F]exendin f) and [^{18}F]FDOPA g) during imaging at 5 months post-implantation in cohort-2, each genotype separate. A single *KCNJ11*$^{+/R201H}$ graft was detected with [^{18}F]FDOPA and is not plotted

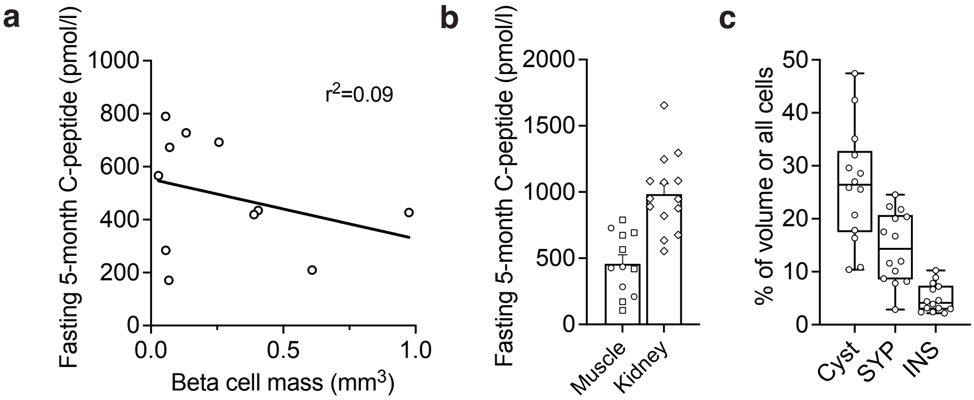

ESM Figure 6, intramuscular graft C-peptide measurements and kidney graft composition:

a) Linear regression of the circulating human C-peptide at 5 months and the sum of the non-cystic, SC-beta cell fraction corrected graft volumes in the two intramuscular grafts in the mice used for the imaging studies. **b**) Fasting C-peptide in the mice used for imaging (muscle, the mice carry two grafts, cohorts pooled) and in the kidney cohort at 5-months post-implantation **c**) Cystic volume as percentage of total graft volume, and the percentage of SYP^+ endocrine cell and INS^+ SC-beta cells out of all cells in the graft (including connective tissue cells)
